# T Cell Activation but Not Polyfunctionality after Primary HIV Infection Predicts Control of Viral Load and Length of the Time without Therapy

**DOI:** 10.1371/journal.pone.0050728

**Published:** 2012-12-07

**Authors:** Andrea Cossarizza, Linda Bertoncelli, Elisa Nemes, Enrico Lugli, Marcello Pinti, Milena Nasi, Sara De Biasi, Lara Gibellini, Jonas P. Montagna, Marco Vecchia, Lisa Manzini, Marianna Meschiari, Vanni Borghi, Giovanni Guaraldi, Cristina Mussini

**Affiliations:** 1 Department of Surgery, Medicine, Dentistry and Morphological Sciences, University of Modena and Reggio Emilia, Modena, Italy; 2 Department of Life Sciences, University of Modena and Reggio Emilia, Modena, Italy; 3 Infectious Diseases Clinics, Azienda Ospedaliero-Universitaria Policlinico di Modena, Modena, Italy; 4 Department of Medical and Surgical Sciences of Mother, Child and Adult, University of Modena and Reggio Emilia, Modena, Italy; University of Cape Town, South Africa

## Abstract

**Objective:**

Immune changes occurring after primary HIV infection (PHI) have a pivotal relevance. Our objective was to characterize the polyfunctionality of immune response triggered by PHI, and to characterize immune activation and regulatory T cells, correlating such features to disease progression.

**Patients and Methods:**

We followed 11 patients experiencing PHI for 4 years. By polychromatic flow cytometry, we studied every month, for the first 6 months, T lymphocyte polyfunctionality after cell stimulation with peptides derived from HIV-1 gag and nef. Tregs were identified by flow cytometry, and T cell activation studied by CD38 and HLA-DR expression.

**Results:**

An increase of anti-gag and anti-nef CD8+ specific T cells was observed 3 months after PHI; however, truly polyfunctional T cells, also able to produce IL-2, were never found. No gross changes in Tregs were present. T lymphocyte activation was maximal 1 and 2 months after PHI, and significantly decreased in the following period. The level of activation two months after PHI was strictly correlated to the plasma viral load 1 year after infection, and significantly influenced the length of period without therapy. Indeed, 80% of patients with less than the median value of activated CD8+ (15.5%) or CD4+ (0.9%) T cells remained free of therapy for >46 months, while all patients over the median value had to start treatment within 26 months.

**Conclusions:**

T cell activation after PHI, more than T cell polyfunctionality or Tregs, is a predictive marker for the control of viral load and for the time required to start treatment.

## Introduction

Primary infection with the human immunodeficiency virus type-1 (HIV) is a crucial moment for establishing relationships between virus and host [1], [2], [3]. The high plasma viral load (pVL) causes a relevant and persistent immune activation that can trigger apoptosis [6]–[8], and becomes chronic in the absence of a valid immune response or without efficient antiretroviral therapy. The immune activation present in this phase is recognizable by typical changes [4], such as an increase in activated/memory CD8+ T cells that express CD38, CD45R0, human leukocyte antigen-DR, and high amounts of cell adhesion molecules, and which can represent most part of circulating lymphocytes; a decrease in CD4+ T cells is not always present. High plasma levels of proinflammatory cytokines have been described, along with changes in mitochondrial functionality, augmented tendency to apoptosis and expression of cell death markers (such as CD95) in almost all white blood cells [5], [6], [7]. However, no gross alterations in Vβ T-cell repertoire have been found, and the functionality of the T cell repertoire seems well preserved [8]. In turn, immune activation can promote viral replication, so facilitating the infection of other T cells [9], [10]. Several studies, including those in animal models, where primary infection has been experimentally induced and strictly monitored, showed that a strict correlation exists between immune activation and progression of the infection [11].

During PHI, the appearance of virus-specific cytotoxic T lymphocytes (CTL) coincides with the decay of viral replication, so that patients with a high frequency of HIV-specific CTL display a low pVL and a slow decrease in CD4+ T cell count [12], [13]. A significant direct association between the frequency of CD8+ gag-specific T cells and the length of AIDS-free period has been observed during chronic infection [14]. Specific T helper cells are crucial for the anti-HIV immune response, since they provide help to B and CD8+ cells. A recent study in SIV-infected macaques has shown that depleting CD4+ during PHI worsen the infection [15].

HIV preferentially infects HIV-specific CD4+ lymphocytes [16]. The efficacy of a specific immune response is due to CD4+ and CD8+ T cell clones with multiple effectors functions, such as production of different cytokines and chemokines, activity of costimulatory molecules, capacity to perform degranulation and to express cytotoxic molecules (e.g., perforin) [17], [18]. These cells, defined “polyfunctional”, are present at relatively low frequency in HIV+ patients, but at high frequency in the blood of patients who control the virus, such as long term non progressors (LTNPs) or “élite controllers”, where the presence of HIV-specific polyfunctional CD8+ lymphocytes is associated with spontaneous control of viral replication [19], [20], [21], [22]. Very few data exist on the polyfunctionality of T cells immediately after primary infection [23], and we were interesting in investigating this aspect in a longitudinal manner.

Regulatory T cells (Tregs) have a crucial importance, being a viral reservoir, as shown by the presence of HIV-DNA in resting CD4+ Tregs from patients assuming HAART [24]. However, their role during the infection remains unclear. CD4+ Tregs might be important for the reduction of immune activation after PHI or even in chronic infection [25]. During chronic infection they could cause the deregulation of HIV-specific response [26], so favoring the progression of the infection, and a decrease of such cells has been associated to an increase in CD4+ and CD8+ specific responses to the virus. In chronically infected HIV+ patients, increased proportions, but reduced absolute numbers of circulating Tregs were found, and Treg frequency was largely normalized by HAART [27].

Thus, in order to identify some crucial immunological events that occur during PHI, we analyzed specific response to viral antigens such as gag and nef, regulatory CD4+ T cells, and T cell activation in a group of patients who experienced a well documented PHI, and have been followed for more than 4 years. Our main finding is that T cell activation after PHI, more than T cell polyfunctionality or the presence of Tregs, could be considered as a predictive marker for the viral setpoint and time required to treatment.

## Materials and Methods

### Patients

This longitudinal study enrolled 11 patients (9 males) experiencing PHI, who have been followed by the Infectious Diseases Clinics, University Hospital, Modena (Northern Italy). Median age of patients at enrolment was 37 years (range: 20–56); 7 acquired the infection through homosexual intercourses, 4 were heterosexual. All patients had acute PHI documented by positive ELISA and undefined Western Blot, and were in Fiebig stage III [28]. The date of infection was estimated as about 1 month before undetermined Western Blot or 2 weeks before symptoms onset.

In these patients, clinical events who took patients to the clinical observation were: syphilis (1 case), gonorrhea (1), diarrhea (1), candidiasis (1). Furthermore, one had gallbladder stones, another psoriasis; such pathologies were not considered related to HIV infection. All patients came to the medical observation and HIV testing because they realized to have had a risk because of unprotected sexual intercourses, that occurred few weeks before their first visit.

At enrolment, median plasma viral load (VL) was 305,943 copies/mL, median CD4+ T cell count was 816 cells/µL). Viro-immunological parameters (standard CD4+ T cell count and quantification of VL) were performed in untreated patients up to 48 months from PHI (specifically at 12, 24, 36, 48 months) or up to the start of therapy. Chiron branched-DNA was used for plasma HIV RNA, and a value below 50 copies/mL was considered undetectable. Immunological analyses were performed in the first (M1), second (M2), third (M3), fourth (M4) and sixth (M6) month after infection. The different length of the observation period, during which no patients took antiretroviral therapy, was due to different time of enrollment; the longer period of observation in the survival analysis is due to the fact that during the time required to perform the analyses here described patients continued to be followed.

The study has been conducted according to Declaration of Helsinki principles, and approved by the Modena University Review Board. All patients gave written informed consent for the studies here described, according to the Italian laws.

### Samples

Peripheral blood mononuclear cells (PBMC) were purified from EDTA-treated whole blood using Ficoll gradient [29], and cryopreserved according to standard procedures [30]. Thawed PBMC were immediately divided in two aliquots: the first part was stained for phenotype analysis; cells in the second part were rested at least 4 hours at 37°C, in a 5% CO_2_ incubator, in complete RPMI medium [RPMI 1640 supplemented with 10% heat-inactivated fetal calf serum (FCS), and 1% of each L-glutamine, sodium pyruvate, non-essential amino acids and antibiotics; all obtained from Invitrogen, Carlsbad, CA] before stimulation.

### PBMC stimulation

After resting and washing, 2×10^6^ cryopreserved PBMC were incubated overnight in presence of a pool of 15-mer peptides overlapping by 11 amino acids (obtained through the AIDS Research and Reference Reagent Program, Division of AIDS, NIAID, NIH; final concentration was 2 µg/mL/peptide) spanning the sequence of HIV-1 gag (123 peptides) and nef (49 peptides), consensus sequence B. For each sample 0.5×10^6^ cells were left unstimulated as negative control and for each experiment another 0.5×10^6^ cells were stimulated with 1 µg/mL *Staphylococcus aureus* enterotoxin B (SEB, Sigma-Aldrich, St. Louis, MO) as positive control. All samples were incubated in presence of the secretion inhibitors monensin (2.5 µg/mL; Sigma-Aldrich) and brefeldin A (5 µg/mL; Sigma-Aldrich), the costimulatory monoclonal antibodies (mAb) anti-CD28 (1 µg/mL, R&D Systems, Minneapolis, MN) and anti-CD49d (1 µg/mL, Serotec, Oxford, UK); anti-CD107a mAb conjugated with PE-Cy5 (BD Biosciences, San José, CA) was simultaneously added to detect degranulation [21].

### Flow cytometry analysis

Different mAb directly conjugated with different fluorochromes, obtained from eBioscience (San Diego, CA) (anti-CD154-FITC, anti-IL-2-PE, anti-IFN-γ-PE-Cy7, anti-CD4-APC-Alexa 750, anti-HLA-DR-PE-Cy7, anti-CD38-PE), R&D Systems (anti-CD8-APC) and Serotec (anti-CD3-Alexa 405) were pre-titrated with the appropriate buffer before use to identify the optimal combinations and concentrations [31].

Cells were stained with the LIVE/DEAD Red Stain Kit (Molecular Probes, Eugene, OR) and with different mAb for surface antigens, incubated for 20 minutes at room temperature and washed with PBS containing 5% FBS and 5 mM EDTA. Cells were fixed and permeabilized with the “Cytofix/Cytoperm buffer set” from Becton Dickinson for intracellular cytokine detection or with the “Foxp3 Fixation/Permeabilization” kit from eBioscience prior to quantify the intracellular FoxP3 detection. Samples were finally fixed in PBS+1% paraformaldehyde, kept at 4°C.

A multilaser CyFlow ML flow cytometer (Partec GmbH, Münster, Germany) was used to acquire the samples, and the data were analyzed using FloMax (Partec) and FlowJo v8.8.6 (Tree Star Inc., Ashland, OR, USA) softwares. Single staining and “Fluorescence Minus One” (FMO) controls were performed, and gates defining the positive and negative expression of cell surface antigens were combined by boolean gating strategy, as described [32]. Simplified Presentation of Incredibly Complex Evaluations (SPICE) software (kindly provided by Dr. Mario Roederer, Vaccine Research Center, NIAID, NIH) was used to graphically depict polychromatic flow cytometry data [33]. For T cell function analysis, we put a threshold of 0.02% on the basis of the distribution of negative values generated after background subtraction, with a minimum of 10 events [34].

### Statistical analyses

The time-dependent behavior of T lymphocyte activation, Tregs and gag- or nef-specific responses were analyzed by the nonparametric analysis of variance using the Skillings-Mack test to address the presence of missing data. Tukey-Kramer test was used for pairwise comparisons between the months. Differences were considered statistically significant when p<0.05. Linear regressions were performed to investigate the associations between viro-immunological parameters (CD4+ T cell count and pVL), time without therapy and CyFlow data. Survival analysis and Cox proportional hazards model were also performed. STATA 11 for Mac (College Station, TX) was used for performing statistical analyses and obtaining part of the graphics.

## Results

### PHI cohort and viro-immunological parameters

**Figure 1:**
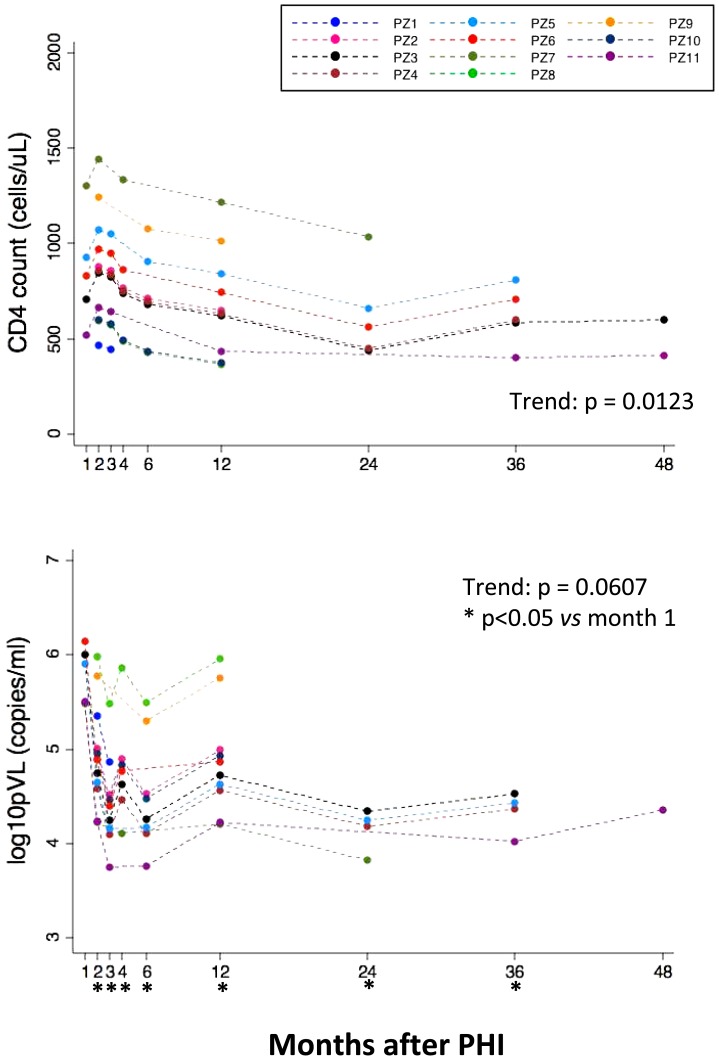
Kinetics of changes in CD4+ T cell count (cell/µL blood, upper panel) and plasma viral load (pVL, number of copies/mL blood, lower panel) after primary HIV infection. Each patients is represented by a different colour.

CD4+ T cell count and pVL were monitored in PHI patients from the first month up to 4 years after infection. It has to be underlined that the number of missing data (in terms of analysis not performed or data not reliable for technical problems), even if carefully considered by the statistical methods we used, was negligible in all cases, *i.e.* <2%. Figure 1, upper panel, shows that CD4+ T cell count increased during the first three months, with a gradual decline in following months (p = 0.0123). In parallel, we observed a decrease in plasma viral load (p = 0.0607) until the onset of a stabilization of plasma viral load by the second month of infection (Figure 1, lower panel). Indeed, at months 2, 3, 4, 6, 12, 24 and 36, viral load was significantly reduced in comparison to M1.

### CD107a expression dominates CD4 gag- and nef- specific response

**Figure 2:**
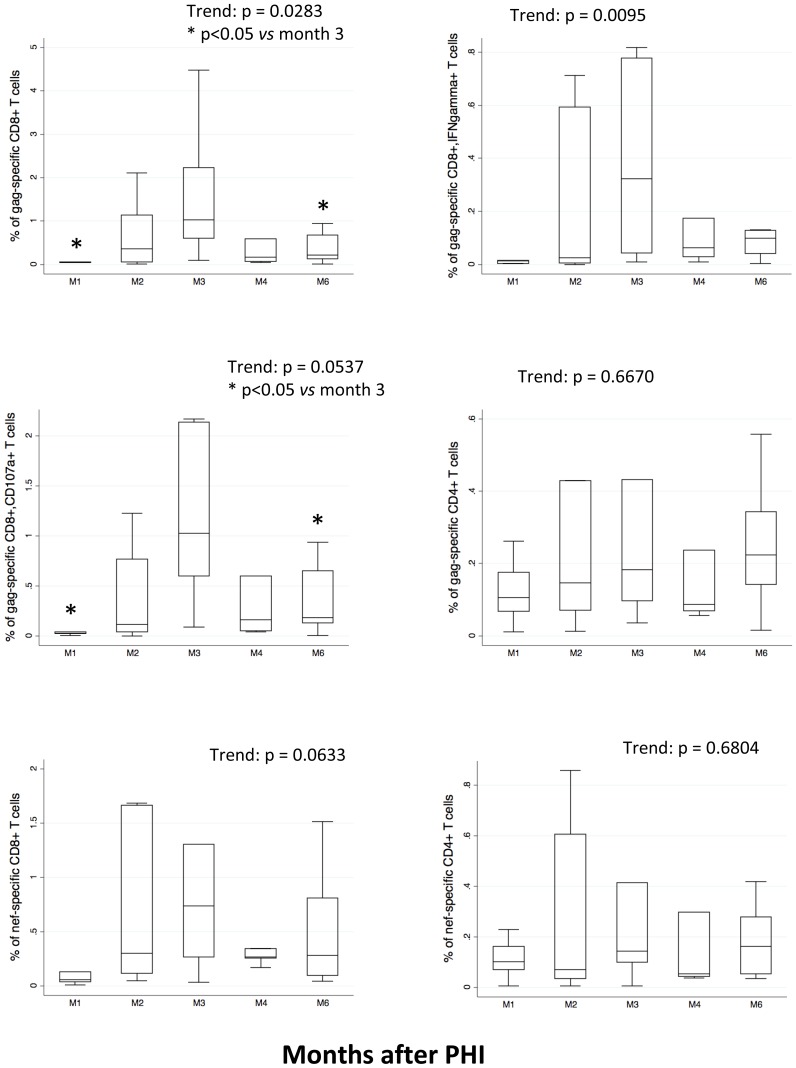
Trends of CD8+ T and CD4+ T cell response to gag- and nef-derived peptides. Boxes indicate median values with 25th and 75th percentiles, whiskers show minimum and maximum. Figure shows the total response to viral peptides, *i.e.*, the sum of all cells positive for at least one of the markers studied (IL-2, IFN-γ, CD154 and CD107a). Patients were studied at month 1 (M1), month 2 (M2), month 3 (M3), month 4 (M4), and month 6 (M6) after PHI. The values of nonparametric analysis of variance (Skillings-Mack, and p values) are reported in figures. Stars in graphs indicate the significant differences of pairwise comparisons between the indicated months, performed by Tukey-Kramer test.

We studied CD4+ and CD8+ T lymphocyte specific polyfunctional response to gag and nef peptides, considering the production or expression of molecules such as IFN-γ, CD107a, CD154 and IL-2. We identified both the “total” response, *i.e.* the sum of all cells positive for at least one marker (that provides the overall “frequency” of responding cells among T lymphocytes), and the “qualitative” response, which describes the contribution of each functional pattern to the total specific response. Figure 2 shows a significant change over time in the percentage of total CD8+ gag-specific cells, and indicates that CD8 response was higher at M3 than at M1 or M6. The same trend was found considering those gag-specific CD8+ T lymphocytes that produced IFN-γ, or those that expressed CD107a. We could not detect any significant variation in the percentage of gag-specific CD4+ T cells, or in the percentages of both nef-specific CD4+ and CD8+ T cells over time.

**Figure 3:**
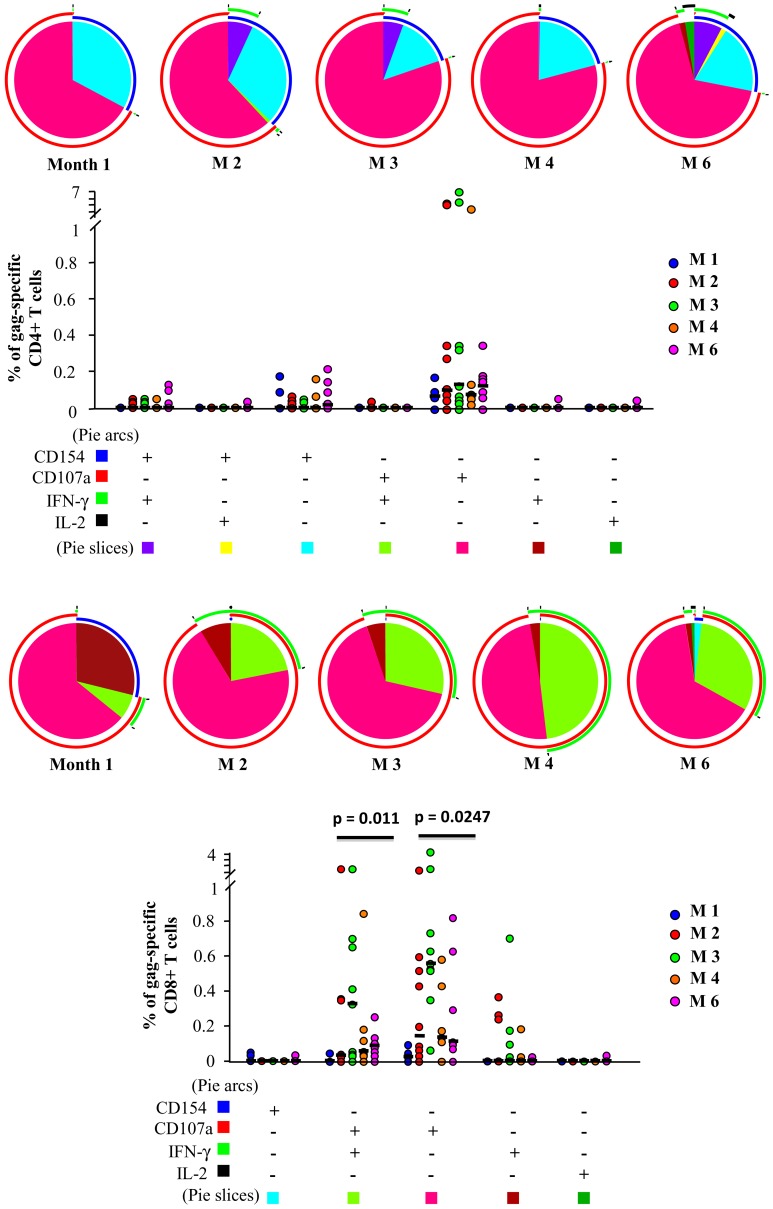
Characterization of the T cell response to gag-derived peptides. Pie charts show the qualitative composition of total gag-specific CD4+ or CD8+ T cell response; each pie slice represents the mean proportion of the total CD4+ T cell response contributed by a single functional pattern, as indicated in the bottom legend. Arcs designed outside the pies represent the fraction of total cells expressing a particular marker, irrespectively of the positive or negative expression of other markers (blue: CD154; red: CD107a; green: IFN-γ; black: IL-2).

Regarding the quality of T cell specific response to gag (Figure 3, upper part), we found that at all the time points >50% of gag-specific CD4+ T lymphocytes were CD107a; only a low percentage was CD154+ or CD154+,IFN-γ+. IL-2 production was detectable only at M6, and in a negligible amount of cells. Figure 3, lower part, shows that gag-specific CD8+ T lymphocytes were predominantly CD107a+, and many of them also produced IFN-γ at all time points. IL-2 production was almost never detected. A significant trend over time was observed in both CD107a+,IFN-γ+ (p = 0.0011) and CD107a single positive (p = 0.0247) CD8+ T cells, with an increase at M2 and M3 and a reduction in the following months.

**Figure 4:**
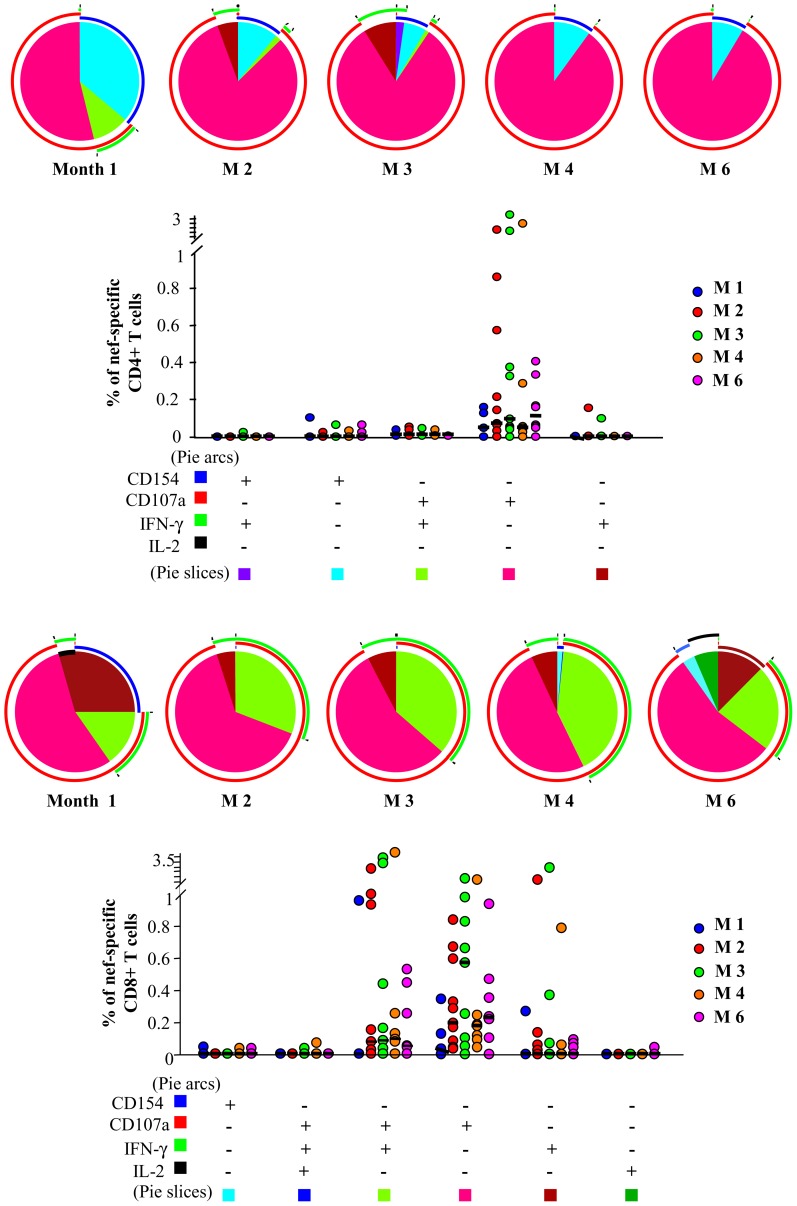
Characterization of the T cell response to nef-derived peptides. Pie charts show the qualitative composition of total gag-specific CD4+ or CD8+ T cell response; each pie slice represents the mean proportion of the total CD4+ T cell response contributed by a single functional pattern, as indicated in the bottom legend. Arcs designed outside the pies represent the fraction of total cells expressing a particular marker, irrespectively of the positive or negative expression of other markers (blue: CD154; red: CD107a; green: IFN-γ; black: IL-2).

Figure 4 (upper part) shows that also nef-specific CD4+ T cell response was characterized a relevant expression of CD107a; only a small amount of cells were able to express CD154 or to produce IFN-γ; IL-2 production was almost never detected. The nef-specific CD8+ T cell response (Figure 4, lower part) was similar to that observed for gag: a large proportion of cells were CD107a+ and/or IFN-γ+, while a negligible amount of cells expressed CD154 or produced IL-2.

### Treg frequency returns to baseline level 6 months after HIV infection

**Figure 5:**
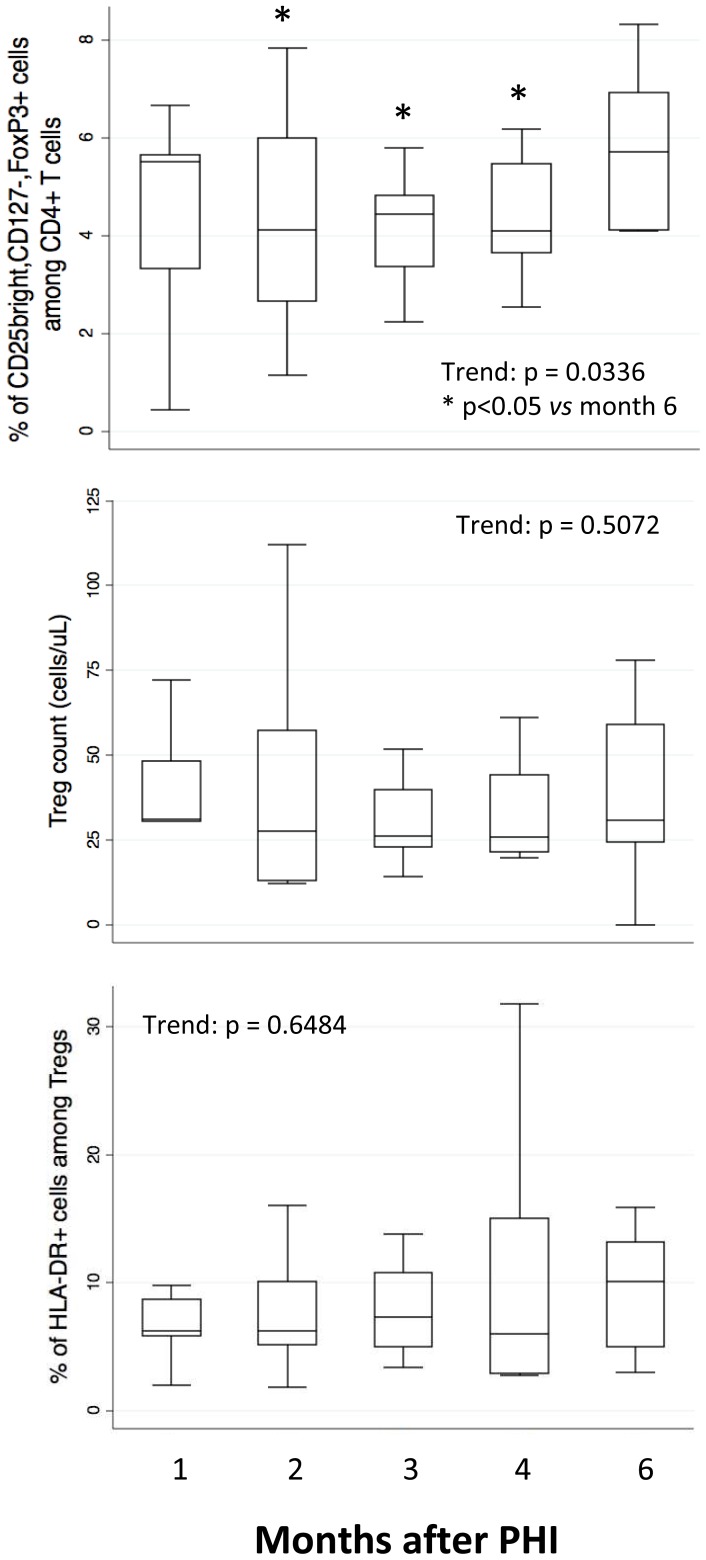
Trends of the Treg frequency (upper panel), absolute number (middle panel) and activated Treg (lower panel), in the patients under investigation. Tregs were considered as those cells that were CD3+,CD4+, positive to FoxP3, highly positive (*i.e.*, bright) to CD25 and negative to CD127. Boxes indicate median values with 25th and 75th percentiles, whiskers show minimum and maximum.

We analyzed the frequency and absolute number of Tregs, defined as CD3+,CD4+,CD25++,CD127−,FoxP3+ cells. As shown in Figure 5, the frequency of CD4+ T cell with regulatory phenotype increased over time (upper panel). However, the absolute number of Treg did not change significantly (middle panel), as well as the amount of Treg showing an activated phenotype (*i.e.*, those expressing HLA-DR, lower panel).

### Trend of T cell activation

**Figure 6:**
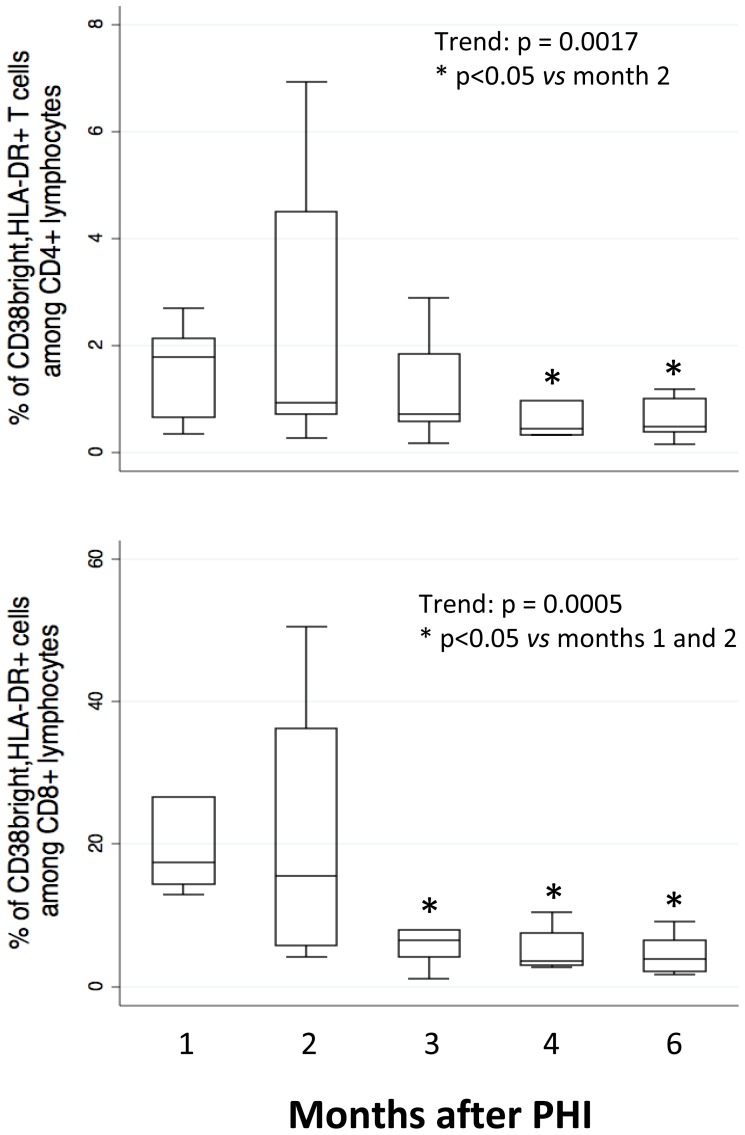
Percentages of activated CD4+ (upper panel) and CD8+ (lower panel) T cells. Activated cells were considered those expressing high levels of CD38 (CD38 bright) and MHC class II (HLA-DR) molecules. Boxes indicate median values with 25th and 75th percentiles, whiskers show minimum and maximum.

The activation of CD4+ and CD8+ T cells was studied taking into account the co-expression of HLA-DR and of high levels of CD38 (CD38^bright^), as described [7]. Figure 6 (upper panel, referred to CD4+, and lower panel, referred to CD8+ T cells) shows a high level of activation at M1 and M2, and a significant decrease in the following period.

### T cell activation after PHI is a predictive marker for viral setpoint and length of the period without therapy

**Figure 7:**
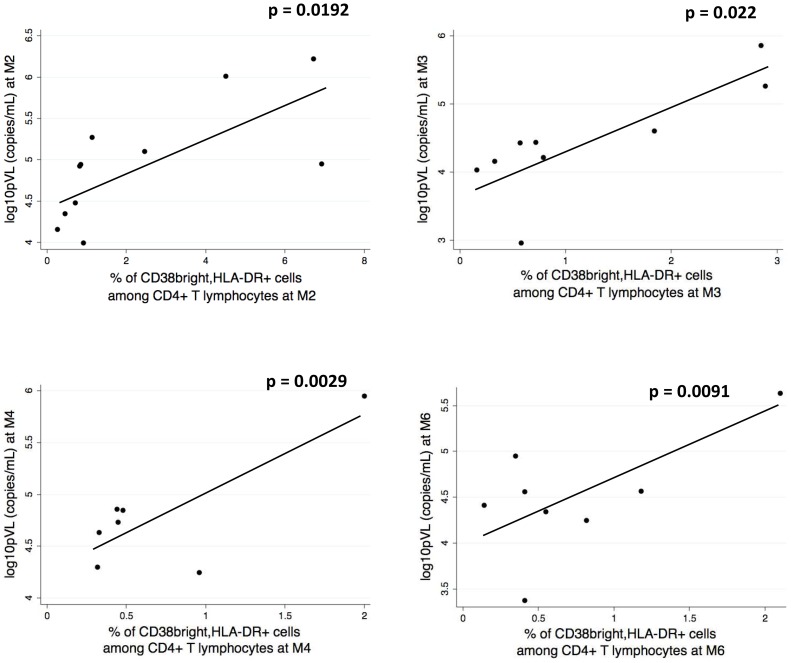
Correlation between the level of CD4+ T cell activation status and pVL levels, at all months analyzed. Activated cells were identified as described in the legend to Figure 6.

**Figure 8:**
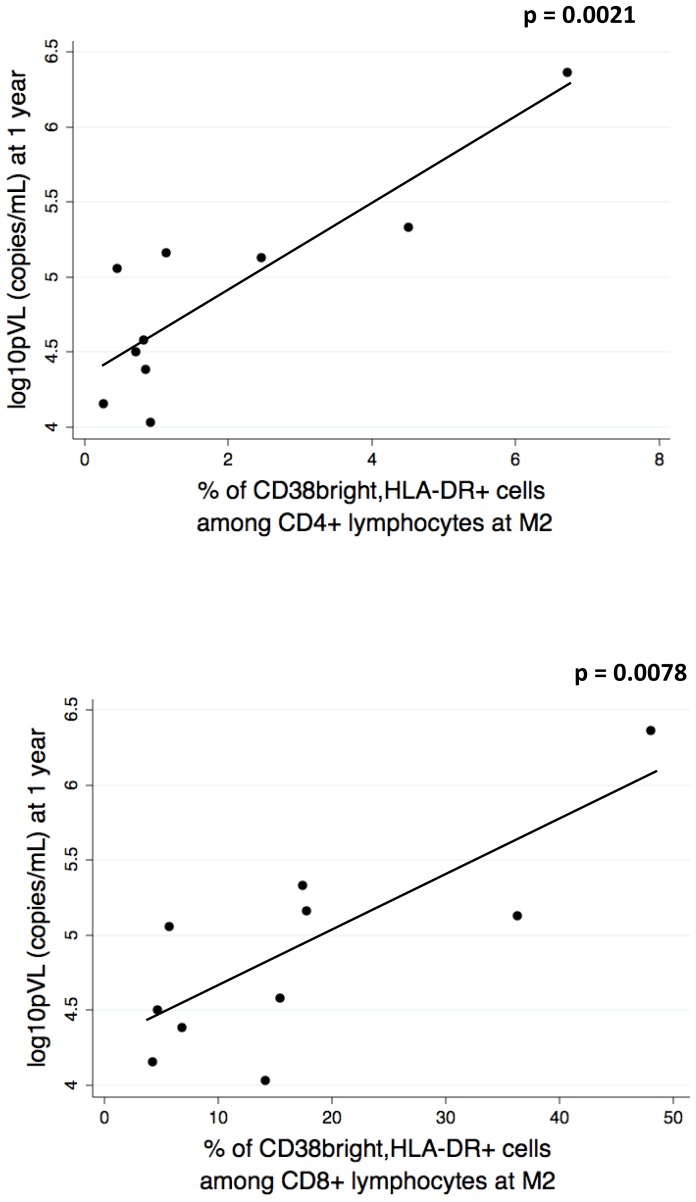
Association between the frequency of activated T cells (either CD4+ or CD8+) measured at M2, and plasma viral load, analyzed 1 year after PHI. Activated cells were identified as described in the legend to Figure 6.

Figure 7 shows that a direct correlation was present between the level of CD4+ T cell activation status and pVL levels, at all months analyzed. As reported in Figure 8, a direct association was then found between the frequency of activated T cells (either CD4+ or CD8+) measured at M2, and plasma viral load, analyzed 1 year after PHI.

**Figure 9:**
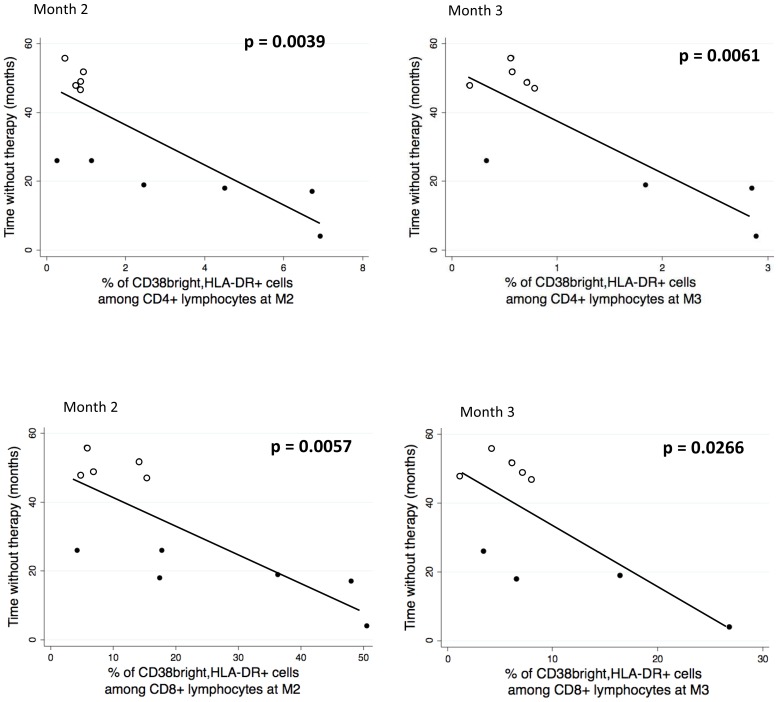
Association between CD4+ or CD8+ T cell activation at M2 and M3 and the length of the period free of therapy. Activated cells were identified as described in the legend to Figure 6.

Finally, an inverse association between CD4+ or CD8+ T cell activation at M2 and M3 and the length of the period free of therapy was also found (Figure 9).

### Activation of CD8+ T cells predicts the length of the period without therapy

We analyzed the role of CD8+ T cell activation in predicting the length of the period without treatment, and performed a “drug free” survival analysis in which we considered the importance of T cell activation in influencing the length of the period that did not require any treatment, i.e. from PHI to the failure of virological control (the time of starting HAART).

**Figure 10:**
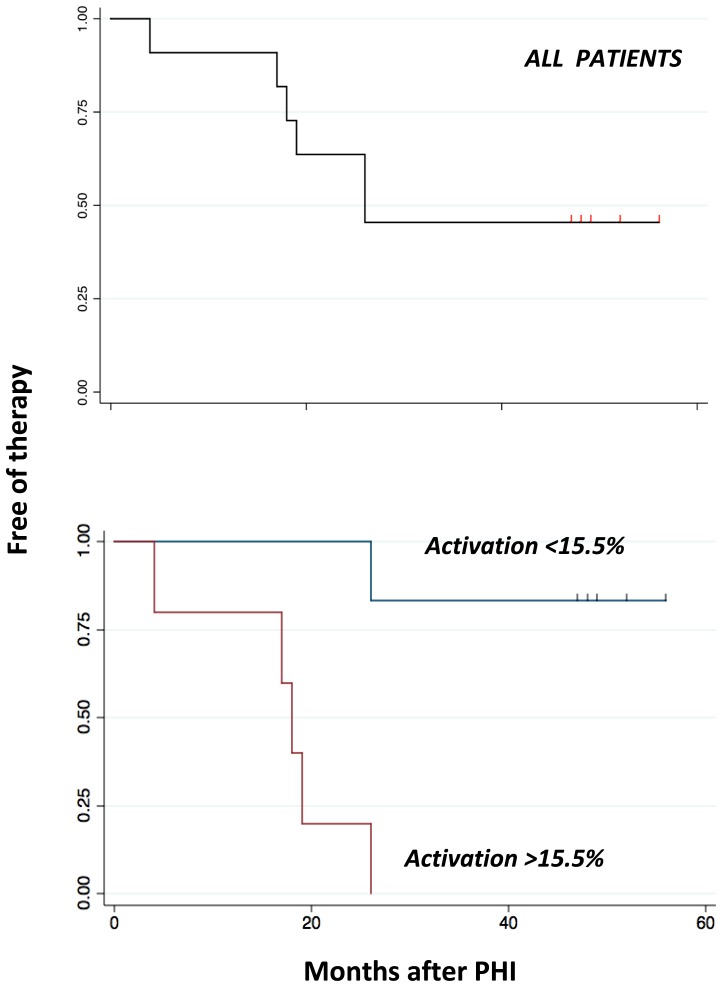
Kaplan-Meier survival curves related to the free-of-therapy period. The survival analysis was performed considering the time elapsed from PHI to the occurrence of failure, defined as the start of antiretroviral therapy. Upper pane, all patients; lower panels, patients with different degree of CD8+ T cell activation.

Figure 10 (upper part) shows time free of therapy of all our patients, performed by Kaplan Meyer analysis: 25% of patients failed (i.e., had to start therapy) within 18 months of HIV infection, and 50% within 26 months. Five out of 11 patients were still out of therapy 48 months after PHI.

Two months after PHI, the median percentage of activated CD8+ T cells was 15.5%, that of CD4+ T cells was 0.9%. By Cox analysis, we found that activation of CD8+ T cells had a significant impact on the risk of starting therapy (Hazard ratio = 1.124 p>|z| = 0.013; 95% Conf. Interval: 1.030–1.232): the increase in one unit of CD8 activation leads to an increase in the instantaneous risk of 2.5% to 23%. As shown in Figure 10 (lower part), we found that all patients with values of activated CD8+ T cells above the median (5 out of 11) had to start therapy within 26 months from PHI. Five out of 6 whose values of CD8+ T cell activation were below the median were still out of therapy for more than 48 months. It is to note that identical results were obtained considering activated CD4+ T cells: 80% of patients with less than the median value remained free of therapy for >48 months, while all patients above the median had to start treatment within 26 months (not shown).

## Discussion

Acute, primary HIV infection has a crucial importance in determining the entire course of the disease, since in this phase the immune activation set point predicts the loss of CD4+ T lymphocytes [2]. Indeed, it has been shown that the level of CD4+ and CD8+ T cell activation during acute infection is able to influence the level of HIV viremia, that the level of CD8+ T cells that express the activation marker CD38 predicts the loss of CD4+ T cells [35], and that not only the reduction of viremia by antiretroviral treatment reduces immune activation and inflammatory phenomena, but also that the reduction of T cell activation by anti-inflammatory drugs can reduce viremia [36]. A strong immune activation, that includes the production of proinflammatory cytokines and rapid destruction of CD4+ T cells that reside in the gut-associated lymphoid tissue, favors massive replication of the virus and its dissemination in lymphoid tissues. In turn, the impaired local cellular immunity favors damages to the gastrointestinal mucosa, allowing the translocation of microbial products, including LPS, which contribute to persistent inflammation through the activation of Toll like receptors.

Furthermore, self-molecules containing the so-called “damage-associated molecular pattern”, or alarmins, that include mitochondrial proteins and mitochondrial DNA [37], can further activate inflammatory pathways and increase the damages [38], [39]. Gaining information on the events that occur during primary infection is thus crucial to find strategies that can either arrest the initial virus spread, or rescue host cells. To better understand the importance of immune activation in determining the course of HIV infection, we have performed a longitudinal study in a group of patients in whom several immune parameters were studied for six months after primary infection, and who were then followed for up to 5 years.

We analyzed both the subtype and magnitude of specific T lymphocytes that respond to the viral proteins gag and nef, and observed an increased level of activity of CD8+ T cells 3 months after infection. CD4+ T cell specific response against HIV peptides did not significantly change during the first 6 months and, as observed in our previous studies on treatment interruption (which could be considered a sort of secondary acute infection) or by others (during acute primary infection) was mainly characterized by cytotoxic features, including the expression of CD107a [21], [40]. The efficacy of anti-HIV specific response has been linked to the “polyfunctionality” of specific clones, *i.e.* to those cells able to exert simultaneously multiple effectors functions. However, in the first months after PHI, we could not find truly polyfunctional CD4+ and CD8+ T cells, *i.e.* those performing 4 functions: indeed, we could not detect significant amounts of cells able to produce IL-2.

The influence and role of Treg during HIV infection remain unclear. Discrepant results have been reported, likely depending on the patient populations, the type and length of treatment, patient's age, the time points analyzed, and the way Tregs were characterized [41], [42], [43]. Most studies showed that during HIV infection Treg increase in frequency but decrease in absolute number, either in blood or in other compartments, likely because of an increased generation, survival or proliferation in the periphery, or because of a different redistribution among tissues [reviewed in 44]. The role played by Tregs during HIV infection is still poorly understood, as two opposing hypotheses have been proposed. A detrimental role of Tregs during HIV infection was suggested based on the evidence that Tregs suppress virus-specific immune responses. Conversely, Tregs could be beneficial by limiting immune activation, thus controlling the availability of HIV targets as well as preventing immune-based pathologies. Recently, it has been shown that untreated, chronically infected patients can display a 2-fold increase in the frequency of Tregs [26]. On the contrary, HIV+ patients defined “élite long-term nonprogressors”, with a documented history of at least 12 years of infection and an undetectable viremia showed had fewer activated Treg [45].

Very few data are available on these cells in the first stages of HIV infection. Tregs could be involved in the regulation of the hyper-activation that occur during PHI, but in our study the frequency and number of Tregs were not correlated to the control of the immune activation, to the disease progression, nor to viro-immunological parameters. Thus, it might be hypothesized that the time required by the virus (or by the inadequate response to the virus) to provoke functional or phenotypic Treg alterations is longer than that we have considered in our study.

Finally, we found high levels of activation in the first two months after primary infection, that decreased over time. Immune activation is a well known important and predictive marker in patients with chronic infection [46], being related to several phenomena, including loss of CD4+ T cells in the gastrointestinal tract and the consequent microbial translocation [47], [48].

We are aware that our study has some limitations, the first of which is the limited number of patients followed for a relatively short time. However, it has to be noted that the enrolment of patients with acute HIV infection is quite difficult, and we could only observe a few cases per year. A second limitation of the study is that the percentage of peripheral blood lymphocytes responding to specific HIV peptides were quite low, and that in some cases the individual variability was quite high. These phenomena, along with the number of patients, were likely responsible for the lack of any statistical significance of parameters related to T cell polyfunctionality.

However, here we show that patients who had a lower frequency of activated CD4+ and CD8+ T lymphocytes in the first 2 months after primary infection could remain much longer without antiretroviral therapy, and confirm the importance of the immune activation set point after primary infection [2]. Since patients with low immune activation could remain out of therapy – and thus were able to maintain a relatively high CD4+ T cell count – for a relatively long period, the identification of this immune parameter has to be considered when clinicians visit patients with primary, acute HIV infection. Finally, it is our opinion that the use of relatively simple flow cytometry methods, based on the simultaneous detection of no more than 3 or 4 parameters at the single cell level, could be more than sufficient to identify this biomarker, whose importance is actually not adequately taken into consideration.
